# Enhanced activation of mechanistic target of rapamycin complex 1 signaling in eruptive xanthomas

**DOI:** 10.1016/j.jdin.2023.07.001

**Published:** 2023-07-21

**Authors:** Yan Huang, Baiping Cui, Yun Bai, Juan Wang, Hanyu Dou, Yanfei Jin, Qian Du, Yongxian Lai, Yeqiang Liu, Xiaolei Ding

**Affiliations:** a Institute of Geriatrics, Affiliated Nantong Hospital of Shanghai University (The Sixth People’s Hospital of Nantong), School of Medicine, Shanghai University, Nantong, China; b Shanghai Engineering Research Center of Organ Repair, School of Medicine, Shanghai University, Shanghai, China; c Shanghai Skin Disease Hospital, School of Medicine, Tongji University, Shanghai, China

**Keywords:** eruptive xanthoma, lipid metabolism, macrophage, mTOR, mTORC1

*To the Editor:* Eruptive xanthomas are benign skin lesions involving excessive lipid deposition in the dermis.^1^ It is characterized by localized or disseminated small, reddish and yellow, erythematous papules or nodules and is a sign of increased risk for metabolic diseases, including cardiovascular diseases and diabetes (Supplementary Fig 1, *A*; available via Mendeley at https://data.mendeley.com/datasets/9r67kdx4mv/1).^2^ Disturbed lipid metabolism, induced by excess caloric intake or a predisposing genetic factor, can lead to an increased level of circulating lipids, mainly triglycerides and cholesterol esters.^2^ The circulating lipids accumulate in the dermis and are taken up by macrophages, which turn foamy in appearance (Supplementary Fig 1, *B*; available via Mendeley at https://data.mendeley.com/datasets/9r67kdx4mv/1). Despite the evidence emphasizing the role of metabolic dysregulation in eruptive xanthomas, the activation and implications of metabolic signaling pathways in the disease remain largely elusive.

The mechanistic target of rapamycin (mTOR), a serine/threonine kinase, exists in 2 distinct complexes, referred to as mTORC1 and mTORC2.^3^ mTORC1controls cell growth and metabolism by sensing multiple extracellular and intracellular nutrients, growth factors, and cellular energy. When mTORC1 is activated, it phosphorylates eukaryotic translation initiation factor 4E binding protein 1 (4E-BP1) and S6 kinase. Phosphorylated 4E-BP1 releases the eukaryotic translation initiation factor 4E, and S6 kinase further phosphorylates S6 to promote protein translation. Activated mTORC2 mainly affects cell survival and proliferation through phosphorylating downstream mediators, such as Akt (Ser473). Aberrant activation of mTOR signaling has been linked to numerous cancers and metabolic diseases.^3^ Here, we assessed the activation of mTOR signaling in eruptive xanthomas.

To determine mTOR signaling activation in eruptive xanthomas, we performed immunohistochemical staining with antibodies against phosphorylated S6 (Ser240/244), 4E-BP1 (Thr37/46), and Akt (Ser473) in eruptive xanthomas (diagnosed by our dermatology clinic) and healthy skin as a control. In line with our previous observations,^4^ phosphorylated S6 was primarily detected in the granular layer of healthy skin. In contrast, it was markedly increased in the eruptive xanthoma dermis compared with healthy skin (Supplementary Fig 2, *A*; available via Mendeley at https://data.mendeley.com/datasets/9r67kdx4mv/1). Phosphorylated 4E-BP1 displayed a similar pattern of staining as the phosphorylated S6 (Supplementary Fig 2, *B*; available via Mendeley at https://data.mendeley.com/datasets/9r67kdx4mv/1). Quantification of stained signals showed that both phosphorylated S6 and 4E-BP1 were significantly increased in eruptive xanthoma dermis compared with healthy controls (Fig 1, *A* and *B*). By contrast, phosphorylated Akt, the best characterized target of mTORC2, was nearly undetectable in either healthy skin or lesional eruptive xanthomas (Supplementary Fig 2, *C*; available via Mendeley at https://data.mendeley.com/datasets/9r67kdx4mv/1). Collectively, these data demonstrate that mTORC1 signaling is specifically hyperactivated in eruptive xanthomas.

**Fig 1:**
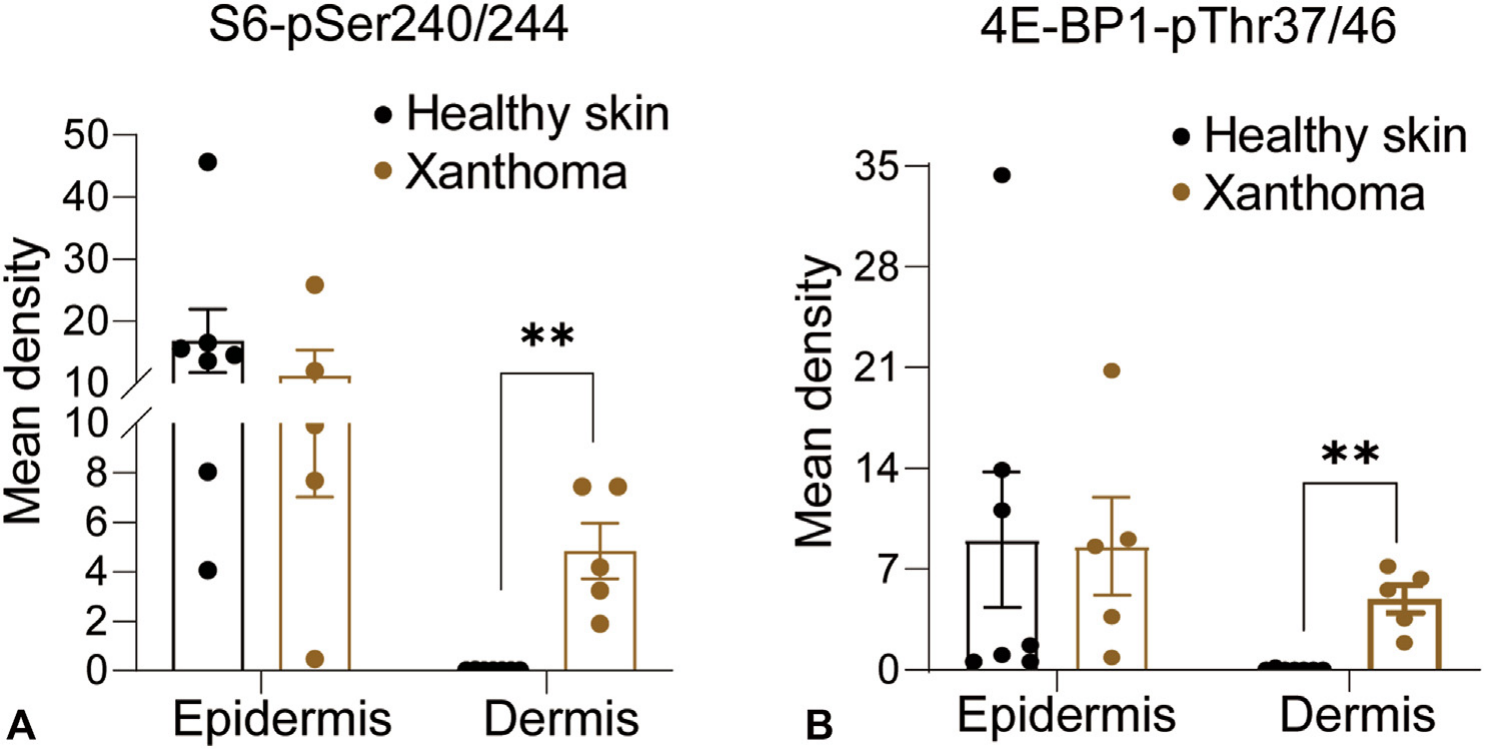
Mechanistic target of rapamycin complex 1 (mTORC1) signaling activation is upregulated in eruptive xanthomas. Areas that stained positive with indicated antibodies were quantified and shown. Each dot represents a different patient. Data represent mean ± SEM; nonpaired *t* test was used to calculate *P* values. ∗∗*P* < .01. *4E-BP*, Eukaryotic translation initiation factor 4E binding protein; *pSer*, phosphorylated serine; *pThe*, phosphorylated threonine.

These findings suggest that mTORC1 signaling may play a causal role in eruptive xanthomas, and mTORC1 signaling can be a novel therapeutic target for eruptive xanthoma treatment. However, the mechanism underlying enhanced mTOR activation in eruptive xanthomas remains to be elucidated. Theoretically, the availabilities of nutrients, growth factors, and cytokines are all able to induce mTORC1 activation. Interestingly, recent studies demonstrated that lysosomal cholesterol can directly activate mTORC1 signaling^5^ and that the hyperactivated mTORC1 signaling might be induced by overloaded lipids. Further dissecting the underlying mechanisms will not only help understand the pathogenesis of the disease but also provide a novel treatment.

## Conflicts of interest

None disclosed.
